# Identification and Characterization of a Cold-Active Phthalate Esters Hydrolase by Screening a Metagenomic Library Derived from Biofilms of a Wastewater Treatment Plant

**DOI:** 10.1371/journal.pone.0075977

**Published:** 2013-10-08

**Authors:** Yiying Jiao, Xu Chen, Xin Wang, Xuewei Liao, Lin Xiao, Aijun Miao, Jun Wu, Liuyan Yang

**Affiliations:** 1 State Key Laboratory of Pollution Control and Resource Reuse, Department of Environmental Biology, School of the Environment, Nanjing University, Nanjing, People’s Republic of China; 2 Center for Analysis and Testing, Nanjing Normal University, Nanjing, People’s Republic of China; Universidad Autónoma del estado de Morelos, Mexico

## Abstract

A cold-active phthalate esters hydrolase gene (designated *dphB*) was identified through functional screening of a metagenomic library derived from biofilms of a wastewater treatment plant. The enzyme specifically catalyzed the hydrolysis of dipropyl phthalate, dibutyl phthalate, and dipentyl phthalate to the corresponding monoalkyl phthalate esters at low temperatures. The catalytic triad residues of DphB were proposed to be Ser159, Asp251, and His281.

## Introduction

Phthalate esters (PEs) are a category of toxic organic compounds that wildly used as additives or plasticizers (softeners) in the manufacture of plastics [1]. Since PEs are not chemically bound to the host polymers, they have the tendency to leach slowly from the host matrix and migrate into the environment. Therefore they have been detected in various environments, such as sediment, natural bodies of water, soils, and even in the atmosphere [2]–[5]. In recent years, PEs have become great environmental concerns globally because of their suspected carcinogenic, estrogenic, and endocrine-disrupting properties [6]–[10]. Short chain (C_1_–C_4_) alkyl esters of phthalates are shown to be more toxic because of their high solubility compared to longer chain homologs [11]. The US Environmental Protection Agency and a number of its international counterparts have already classified the most common PEs as priority environmental pollutants [12]–[14].

The microbial degradation of PEs has been intensively researched during the last 40 years and the biochemical mechanisms underlying the sequential hydrolysis of PEs to parent compound phthalate (PTH) have been elucidated from some well-studied strains. Two distinct dialkyl PEs hydrolases, respectively, from *Acinetobacter* sp. M673 [15] and *Micrococcus* sp. YGJ1 [16], have been reported to catalyze the hydrolysis of one ester bond of dialkyl PEs to form the corresponding monoalkyl PEs. And three distinct monoalkyl PEs hydrolases, respectively, from *Micrococcus* sp. YGJ1 [17], *Gordonia* sp. P8219 [18], and *Rhodococcus jostii* RHA1 [19], have been reported to catalyze the hydrolysis of the ester bond of monoalkyl PEs to form PTH, which is less toxic than its mono−/di- esters [20]. All these hydrolases are characterized by high catalytic efficiency at moderate temperatures from 25 to 45°C. Here, we described a cold-active hydrolase from a metagenomic fosmid library obtained from the biofilms of a PEs (mainly dibutyl phthalate (DBP)) wastewater treatment plant. This enzyme displayed specific dialkyl PEs hydrolase activity toward three esters, commonly used in the plastic industry, dipropyl phthalate (DPrP), DBP, and dipentyl phthalate (DPP) at low temperatures, with an optimum temperature of 10°C.

## Materials and Methods

### Chemicals

All dialkyl esters and monoalkyl esters were purchased from Sigma-Aldrich (USA) or TCI (Tokyo, Japan). Other chemical reagents used in this study were all of analytical grade and obtained from Shanghai Sangon Biological Engineering Technology & Service Co., Ltd., China. The Meta-G-Nome™ metagenomic DNA isolation kit and the CopyControl™ fosmid library production kit were purchased from Epicentre Biotechnologies (Madison, WI). The large-construct kit for fosmid isolation was obtained from QIAGEN. Other enzymes and kits necessary for DNA manipulations were purchased from Takara Biotechnology. The Superdex 200 HR 10/30 column was purchased from Amersham Bioscience. The ultra-15 centrifugal filter unit with ultracel-5 regenerated cellulose membrane (5-kDa cutoff size) was purchased from Amicon.

### Source of Biofilms

No specific permissions were required for the PEs wastewater treatment plant (Nanjing, China) where the biofilms were collected. Our studies did not involve any endangered or protected species. The biofilms used in this experiment were collected from a membrane bio-reactor, which was well-performed in the winter (at 10°C) in a wastewater treatment plant of a DBP producing chemical industry (Nanjing, China), with nearly 15 mg L^−1^ DBP in influent and 0.35 mg L^−1^ DBP in effluent. The biofilms were stored in a sterile plastic bag and kept at 4°C for 3 days before experiments.

### Degradation of PEs at 10°C by the Biofilms

The degradation of dimethyl phthalate (DMP), diethyl phthalate (DEP), DPrP, DBP, DPP, dihexyl phthalate (DHP), and diheptyl phthalate (DHpP) was carried out in mineral salt medium (SM) containing (per liter) 1.0 g of NH_4_NO_3_, 1.0 g of NaCl, 1.5 g of K_2_HPO_4_, 0.5 g of KH_2_PO_4_, and 0.1 g of MgSO_4_. The pH of SM was adjusted to seven with HCl or NaOH and then sterilized by autoclaving for 15 min at 121°C. 2 g of biofilms was added to a 1 L Erlenmeyer flask containing 300 ml of liquid SM plus 0.1 mM of each PEs. Each flask was incubated at 10°C on a rotary shaker. Appropriate controls containing SM medium plus 0.1 mM of each PEs were prepared simultaneously. Aliquots (2 ml) were taken out periodically and the amount of each substrate was determined by high-performance liquid chromatography/mass spectrometry (HPLC/MS) analysis.

### Metagenomic Fosmid Library Production and Screening

Metagenomic DNA was extracted from the biofilms using the Meta-G-Nome™ DNA isolation kit. A fosmid library (average insert size, approximately 40 kb) was constructed via pCC 1FOS™ vector using the CopyControl™ fosmid library production kit then plating on plating *Escherichia coli* EPI300-T1^R^ strain, which represented 4×10^8^ bp of metagenomic DNA. The library was screened for colonies displaying dialkyl PEs hydrolytic activity by hydrolysis of DBP on Luria-Bertani (LB) agar plates (1.0% NaCl, 1.0% tryptone, 0.5% yeast extract, and 1.5% agar) plus 1.5 mM DBP (a stock solution of 50 mM in dimethyl sulfoxide), and chloramphenicol (17 µg ml^−1^), and incubated at 37°C for 3 days and then 10°C for 5 days. The disappearance of DBP along with production of monobutyl phthalate (MBP) may cause well visible transparent halos on the agar plates [15]. Grown colonies were picked and purified from those having formed a clear halo around them. These cells were then cultured in liquid LB medium at 37°C overnight and harvested by centrifugation for 5 min at 12,000×g at 4°C. Cells were resuspended in an equal volume of SM medium plus 0.1 mM DBP. Cell suspensions were incubated at 37°C with shaking. The ability to transform DBP was further tested by HPLC/MS analysis. Boiled cell suspension was used as control.

### Subclone Library Production and Screening

Fosmid DNA of selected colonies was isolated as templates by the large-construct kit (QIAGEN) and the specific polymerase chain reaction (PCR) amplifications of the reported DBP hydrolase gene [15] were first performed by the primers P1 (5′-ATG AAC GAC GGC GCC ACT CGT TAT-3′) and P2 (5′-TCA TGC TGC GCC GTT AGC TTC GGC-3′). To some colonies failed to be cloned the DBP hydrolase gene, a pUC118 subclone library was then constructed for identification of the corresponding dialkyl PEs hydrolase gene(s). The fosmid DNA from these colonies was partially digested by the restriction enzyme *Sau*3AI. These 4- to 8-kb fragments were recovered by a DNA purification kit (TaKaRa) and ligated into pUC118 previously digested with *Bam*HI and treated with calf intestinal alkaline phosphatase (TaKaRa). The ligation product was then transformed into *E. coli* DH5α cells. The resulting bacterial suspension was spread onto LB-agar medium plus 100 µg ml^−1^ ampicillin and 1.5 mM DBP (a stock solution of 50 mM in methanol), and incubated at 37°C for 3 days and then 10°C for 3 days. Grown colonies were also picked and purified from those having formed a clear halo around them, showing disappearance of DBP from this zone. The ability to transform DBP was further tested by HPLC/MS analysis as described above.

The inserted fragment in the transformant was sequenced at Takara Biotechnology Co. Ltd. Nucleotide and deduced amino acid sequence analyses were performed using Omiga software. BlastN and BlastP were used for the nucleotide sequence and deduced amino acid identity searches (www.ncbi.nlm.nih.gov/Blast), respectively.

### Expression and Purification of the Recombinant DphB

The *dphB* gene was amplified by PCR using primer pairs P_F_ to which was added a *Kpn* I site (underlined) (5′-GG GGT ACC ATG AAC GAC GGC GCC ACT CGT TAT ACC-3′) and P_R_ to which was added a *Hin*d III site (underlined) (5′-CCC AAG CTT TGC TGC GCC GTT AGC TTC GGC GAC-3′), and ligated into pET29a at the *Kpn* I and *Hin*d III sites. *E. coli* BL21(DE3) harboring the resulting plasmid pET29a-*dphB* was grown in LB at 37°C in the presence of 50 µg ml^−1^ of kanamycin to an OD_600_ of 0.5, at which 1.0 mM of isopropyl-β-D-thiogalactopyranoside (IPTG) was added to induce gene expression. After induction at 15°C for 8 h, cells were harvested, re-suspended in 10 mM potassium phosphate buffer (pH 7.5), and disrupted by sonication. After centrifugation at 15,000×g at 4°C for 30 min, the supernatant was collected and further purified by a 2-ml volume of NTA-Ni^2+^ agarose (Qiagen) at 4°C. His-tagged target protein was allowed to bind to the resin in 10 mM potassium phosphate buffer (pH 7.5) plus 0.5 M sodium chloride and 10 mM imidazole, and then was eluted by 10 mM potassium phosphate buffer (pH 7.5) plus 0.5 M sodium chloride and 500 mM imidazole. The following purification was then performed by size exclusion chromatography on a Superdex 200 HR 10/30 column (Amersham Bioscience) equilibrated with 10 mM sodium phosphate buffer (pH 7.5) at a flow rate of 0.5 ml min^−1^. The purified recombinant DphB was concentrated with Amicon Ultra-15 centrifugal filter unit with ultracel-5 membrane (Millipore, MA) and stored in 10 mM sodium phosphate buffer (pH 7.5) plus 1 mM 2-mercaptoethanol and 10% glycerol at 4°C. The protein concentration was quantified by the protein assay kit with bovine serum albumin as a standard.

### Determination of Molecular Mass and Isoelectric Point (pI) of the Recombinant DphB

The molecular mass of the denatured recombinant DphB was estimated by sodium dodecyl sulfate-polyacrylamide gel electrophoresis (SDS-PAGE) using a broad-range molecular weight protein standard. Proteins were visualized after Coomassie brilliant blue R-250 staining. The exact native molecular mass of the recombinant DphB was determined by matrix-assisted laser desorption ionization-time of flight mass spectrometry (MALDI-TOF-MS). The pI of the recombinant DphB was estimated by gel isoelectric focusing (IEF) using a precast Ampholine PAGplate (Amersham Bioscience, Uppsala, Sweden) and IEF standards (Amersham Bioscience).

### Recombinant DphB Characterization

Optimal temperature and pH of the recombinant DphB were determined by incubation of enzyme (0.65 µg ml^−1^ of protein, final concentration) in 10 mM sodium phosphate buffer with 0.2 mM DBP as the substrate for 3 min at a pH range of 5.0 to 10.0 and a temperature range of 4 to 70°C. The reaction was stopped by the addition of a double volume of methanol, and the residual substrate was quantified using HPLC/MS. As a non-treatment control, the same operation was performed but without enzyme addition. For estimates of thermal stability, the recombinant DphB was pre-incubated at a temperature range of 30 to 60°C for 10 to 60 min, and then, the residual activity was determined in 10 mM sodium phosphate buffer (pH 7.5) with 0.2 mM DBP as the substrate for 3 min at 10°C as described above.

The substrate specificity of the recombinant DphB was investigated with C_1_–C_7_ chain dialkyl PEs (dimethyl phthalate (DMP), diethyl phthalate (DEP), DPrP, DBP, DPP, dihexyl phthalate (DHP), and diheptyl phthalate (DHpP)) and monoalkyl PEs (monomethyl phthalate (MMP), monoethyl phthalate (MEP), monopropyl phthalate (MPrP), monobutyl phthalate (MBP), monopentyl phthalate (MPP), monohexyl phthalate (MHP), and monoheptyl phthalate (MHpP)). The hydrolysis was initiated by the addition of enzyme (0.5 to 5 µg ml^−1^ of protein, final concentration) in 10 mM sodium phosphate buffer (pH 7.5) with each substrate at a concentration range of 0.05 to 0.6 mM at 10°C for 2 to 10 min, and was stopped by the addition of a double volume of methanol. The samples were analyzed by HPLC/MS. One activity unit was defined as the amount of enzyme required to catalyze the hydrolysis of 1.0 µmol of substrate per min at 10°C. At least three independent determinations were performed for each kinetic constant. The substrate-free assay system was also used as blank simultaneously. The kinetic parameters were calculated from nonlinear regression data analysis using SigmaPlot Version 8.0 software (SPSS Inc.).

### HPLC/MS Analytical Methods

A double volume of methanol was added to the assay solution and the mixture was vortexed for 1.0 min. After centrifugation at 12,000×g for 5.0 min, the supernatant was collected and analyzed by an Agilent 1290/6460 HPLC/MS spectrometry system with electrospray ionization (ESI) under positive-ion ionization conditions. The ESI-MS conditions were optimized as follows: drying gas temperature, 250°C; drying gas flow (nitrogen), 8 L min^−1^; nebulizer gas pressure (nitrogen), 35 psi; capillary voltage, 4,000 V; sheath gas temperature, 300°C; sheath gas flow, 10 L min^−1^; and nozzle voltage, 400 V. HPLC separation was performed on an Eclipse XDB-C18 column of 4.6×250 mm, 5.0 µm particle size (Agilent Technologies). The mobile phase was 5% (vol) H_2_O containing 0.1% formic acid in methanol with a flow rate of 0.8 ml min^−1^. Positive ions were acquired in full scan mode in the range of m/z 50–600 molecular mass units for identification within a 1-s scan time interval.

### Site-directed Mutagenesis

Mutagenesis of DphB was performed using the principle of the MutanBEST kit (TaKaRa), according to the provided protocol. All of the nucleotide sequences of mutants were sequenced and confirmed at Takara Biotechnology Co. Ltd. Four amino acid residues (Ser159, Asp251, Asp255, and His281) of DphB were converted to Ala residues, respectively. The oligonucleotides used were as follows: Ser159Ala-F (5′-GTC CTG TCC GGC GAA *GC*C GCG GGT-3′), Ser159Ala-R (5′-AAG CCC CGT GCA GGC GAT ATT GTC-3′); Asp251Ala-F (5′-ACG GCG GGA CTC G*C*T CCC TTG CGC-3′), Asp251Ala-R (5′-GAT CAG CAG CGT CGG CGG CAG GTC-3′); Asp255Ala-F (5′-GAT CCC TTG CGC G*C*T CAG GGC CGC-3′), Asp255Ala-R (5′-GAG TCC CGC CGT GAT CAG CAG CGT-3′); His281Ala-F (5′-AAG GGC AAT ATC *GC*C GGC TAC ATC-3′), His281Ala-R (5′-TGC CTC GCG ATA GGT CGT CGG CAC-3′). The nucleotide sequence of the each mutant was amplified using the primers P_F_ and P_R_ and cloned into plasmid pET29a. The resulting plasmid was transformed into *E. coli* BL21. The expression and purification of the recombinant DphB mutants were performed as described above. The substrate specificity of the DphB mutants was also investigated towards DBP as described above.

### Circular Dichroism (CD) Spectra

CD spectra were measured with a JASCO J-720 spectropolarimeter at room temperature. The cells used were 1 and 0.1 cm light paths for wavelengths of 250–320 and 190–250 nm, respectively. 0.3–0.4 mg ml^−1^ of enzymes in 50 mM potassium phosphate buffer (pH 7.5) were used.

### Nucleotide Sequence Accession Numbers

The *dphA* and *dphB* gene sequences obtained in this study have been deposited in the GenBank database under accession numbers KC438415 and KC438416, respectively.

## Results and Discussion

### Degradation of PEs at 10°C by the Biofilms

**Figure 1:**
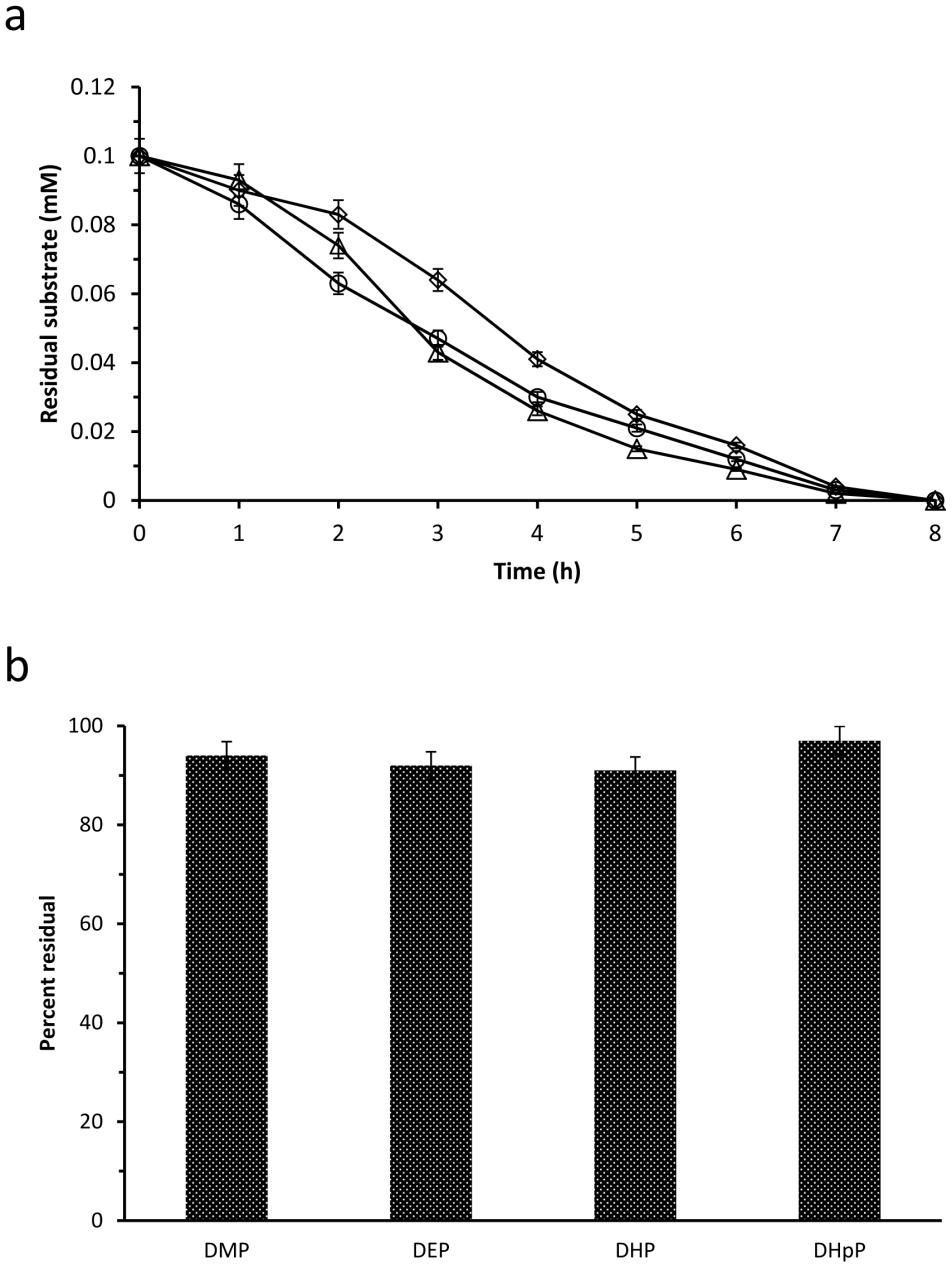
Degradation of seven C_1_–C_7_ chain dialkyl PEs by the biofilms at 10°C. (a), The residual amount from degradation of C_3_–C_5_ chain dialkyl PEs (DPrP, DBP, and DPP) by the biofilms at 10°C; The initial concentration of each dialkyl PE was 0.1 mM; (b), Relative residual amount from degradation of the shorter chain (C_1_–C_2_) esters (DMP and DEP) and the longer chain (C_6_–C_7_) esters (DHP and DHpP) by the biofilms at 10°C; The initial concentration of each dialkyl PE was 0.1 mM and the residual amount was calculated as a percentage of the degraded amounts of dialkyl PEs; Three replicates were conducted in this study; the error bars indicated standard deviations.

The biofilms were tested for their ability to degrade C_1_–C_7_ chain dialkyl PEs containing DMP, DEP, DPrP, DBP, DPP, DHP, and DHpP respectively at 10°C. During the period of incubation, the biofilms showed strong degrading activity to the medium chain (C_3_–C_5_) esters, with completely degradation of DPrP, DBP, and DPP in 8 h (Figure 1a). A small amount of the corresponding monoalkyl PEs (MPrP, MBP, and MPP) and parent compound PTH appeared in the medium during the first 3 h, but rapidly disappeared in the prolonged incubation (data not shown). These results suggested that the initial degradation process by the biofilms was the sequential hydrolysis of the ester bonds of C_3_–C_5_ chain dialkyl PEs to form PTH, and PTH could be degraded continuously by the biofilms. On the other hand, the biofilms showed very weak degrading activity at 10°C to the shorter chain (C_1_–C_2_) esters (DMP and DEP) and the longer chain (C_6_–C_7_) esters (DHP and DHpP), with degradation of less than 10% (Figure 1b) substrate during the 8-h assay.

### Cloning and Sequence Analysis of *dphB*

Although the completely degradation of 0.1 mM DBP by the biofilms has been observed at 10°C within 8 h, the isolation of microorganisms responsible for the degradation of DBP at low temperature was failure, suggesting the existence of uncultured cold-adapted microorganisms. In order to further investigate the degradation mechanism, we constructed the metagenomic fosmid library. Three active fosmid colonies were selected, and one dialkyl PEs hydrolase gene (designated *dphA*) was cloned from two weak-active fosmid colonies by PCR strategy. The *dphA* gene, responsible for the hydrolysis of DBP to MBP, shared the same nucleotide sequence with the DBP hydrolase gene from *Acinetobacter* sp. M673 [15].

**Figure 2:**
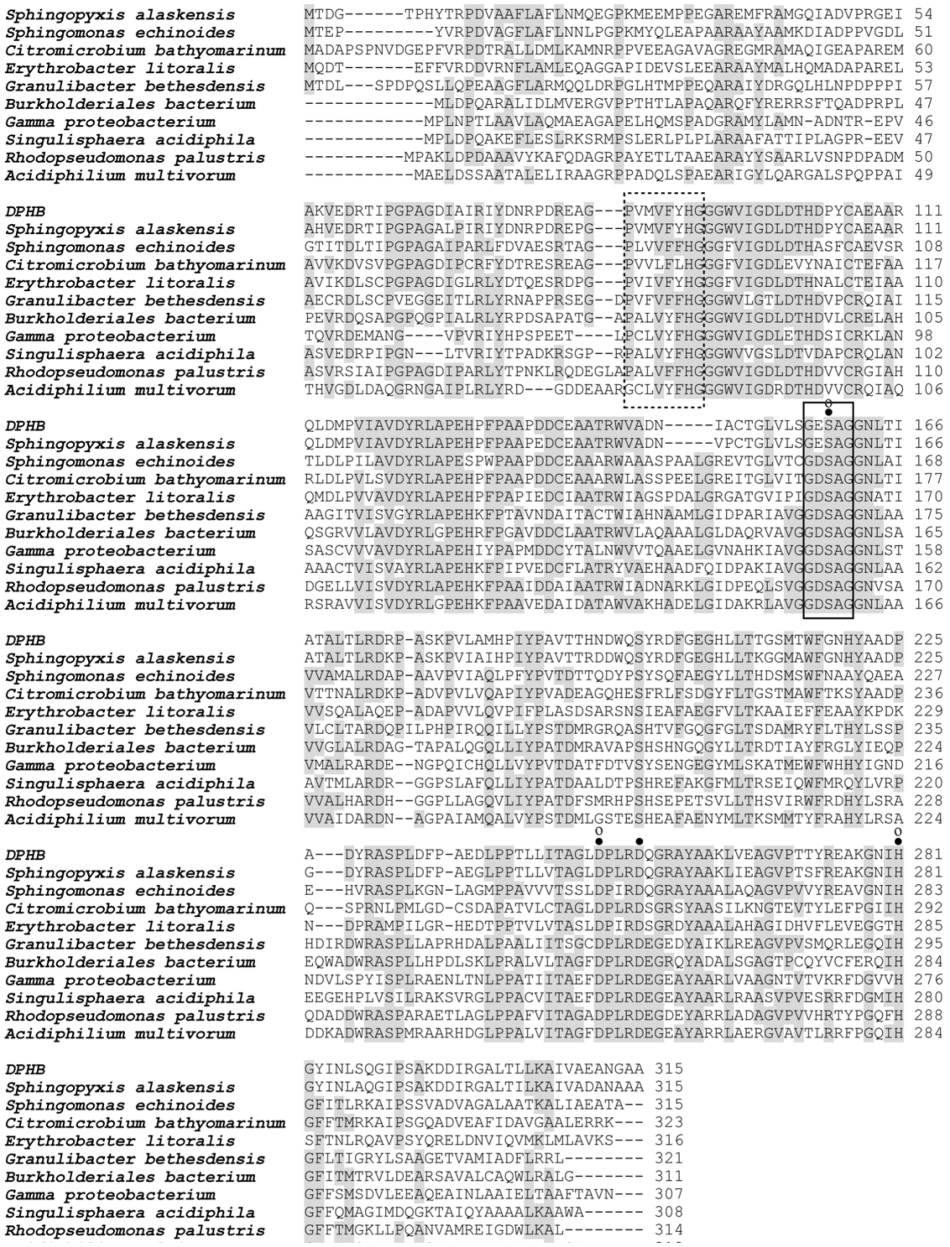
Amino acid alignment of DphB with the hypothetical alpha/beta hydrolase fold-3 from *Sphingopyxis alaskensis* RB2256 (YP_616595), the hypothetical lipases/esterases from *Sphingomonas echinoides* ATCC 14820 (ZP_10341641), *Citromicrobium bathyomarinum* JL354 (ZP_06861760), *Erythrobacter litoralis* HTCC2594 (YP_458304), *Burkholderiales bacterium* JOSHI_001 (ZP_09752441), marine gamma *proteobacterium* HTCC2143 (ZP_01616421), *Singulisphaera acidiphila* DSM 18658 (ZP_09572193), *Rhodopseudomonas palustris* CGA009 (NP_947767), *Acidiphilium multivorum* AIU301 (YP_004283174), and the acetyl esterase of *Granulibacter bethesdensis* CGDNIH1 (YP_746189). A conserved pentapeptide (GXSXG), containing the serine residue of the catalytic triad, was framed by a solid box and a hypothetical conserved oxyanion region (PV-HG) was framed by a dotted box. Hypothetical conserved residues of the catalytic triad were overscribed with an “·”. Confirmed conserved residues of the catalytic triad were overscribed with “o”.

To identify the open reading frame (ORF) corresponding to the dialkyl PEs hydrolase gene in the third strong-active fosmid colony, a pUC118 subclone library was then generated. Several positive transformants those produced a clear transparent halo on the agar plate were screened from approximately 5,000 transformants. The HPLS/MS analysis results showed that these clones were able to completely transform DBP at 10°C while the negative control, *E. coli* DH5α harboring only pUC118, did not at all (data not shown). The sequences analysis (the GeneMark gene prediction tool (http://exon.gatech.edu/GeneMark)) showed that the inserted fragments in these transformants all contained one putative hydrolase-encoding ORF. The ORF (designated *dphB*) was subcloned into the linear vector pMD18-T and used to transform *E. coli* DH5α. Its encoding protein DphB was then confirmed to be the target dialkyl PEs hydrolase. The *dphB* was 948 nucleotides in length with ATG start and TGA stop codons, and encoded a protein of 315 amino acids. Promoter prediction (http://www.fruitfly.org/seq_tools/promoter.html) revealed that there was a promoter-like region located at position 65 to 16 bp upstream from the start codon. A potential ribosome binding site, GGGA, similar to those found in *E. coli*, was found 10 bp upstream from the start codon. No potential signal sequence for secretion was found. Comparative sequence analyses using the BLAST program (http://www.ncbi.nlm.nih.gov/BLAST/) showed that the deduced amino acid sequence of *dphB* was a member of the esterase/lipase superfamily, and had 89% identity with the hypothetical alpha/beta hydrolase fold-3 protein encoded in the genome sequence of *Sphingopyxis alaskensis* strain RB2256 deposited in the NCBI database (GenBank accession no. CP000356), even though it had no overall sequence similarity to any known protein, including other bacterial esterase/lipase. The deduced amino acid sequence contained conserved residues common to esterases/lipases [21], [22]. A GXSXG pentapeptide that formed part of a signature “elbow” near the active site was present in DphB as GESAG at positions 157 to 161. A putative oxyanion binding region that might function to stabilize the oxyanion intermediate in the active site was identified as PV-HG from positions 84 to 91 in the polypeptide [23]–[25] (Figure 2).

### Expression and Purification of the Recombinant DphB

**Figure 3:**
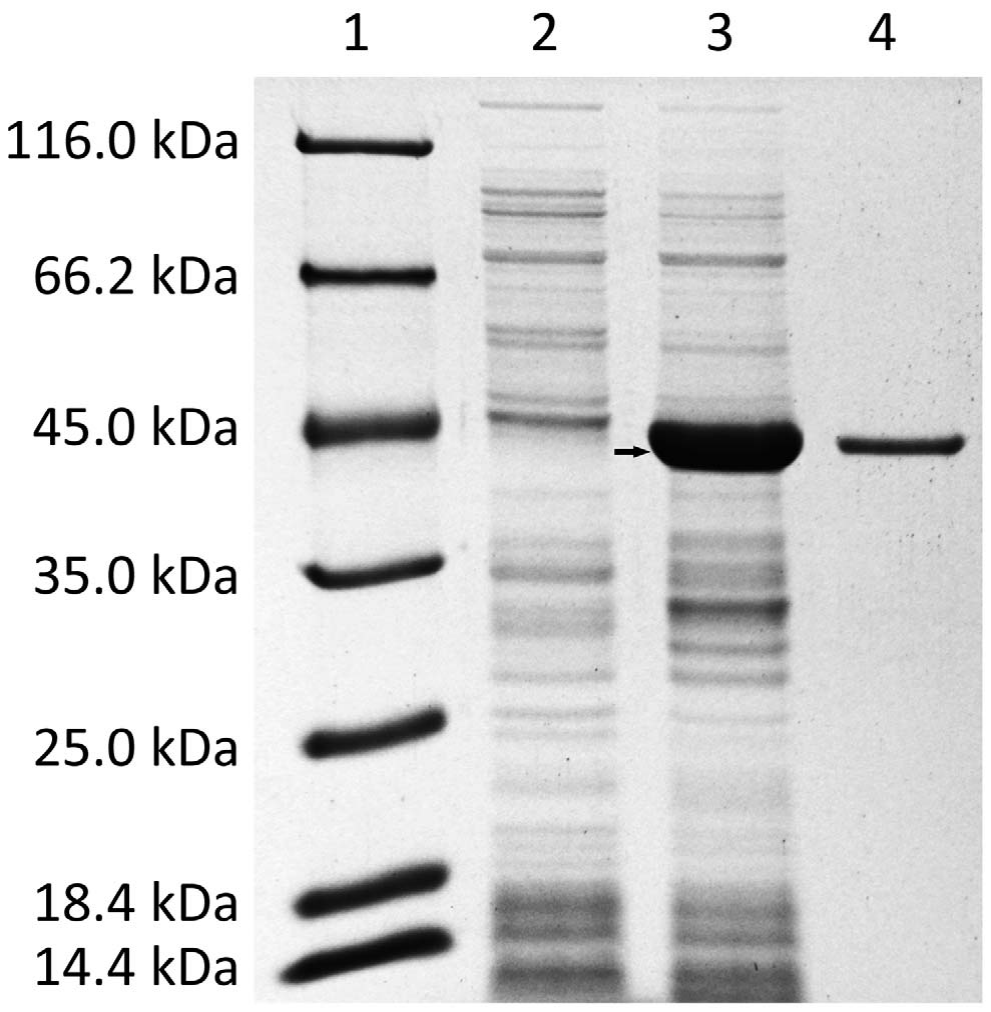
SDS-PAGE analysis of recombinant DphB protein produced in *E. coli* BL21(DE3) harboring plasmid pET29a-*dphB*. Lane 1, protein molecular marker with the sizes 116.0, 66.2, 45.0, 35.0, 25.0, 18.4, and 14.4; lane 2, total proteins of *E. coli* BL21(DE3) harboring plasmid pET29a-*dphB* without IPTG induction; lane 3, total proteins of *E. coli* BL21(DE3) harboring plasmid pET29a-*dphB* with IPTG induction at 15°C for 8 h; the arrow showed the recombinant DphB protein band corresponding to 38 kDa expressed in *E. coli* BL21(DE3) cells; lane 4, 0.5 µg of purified recombinant DphB protein after NTA-Ni^2+^ agarose and size exclusion chromatography steps.

The *dphB* gene was expressed in *E. coli* BL21 (DE3) with or without IPTG, and total proteins were analyzed by SDS-PAGE. Only one induced protein corresponding to 38 kDa was observed (Figure 3), which closed well to the calculated recombinant DphB molecular mass of 37,759 Da. The His-tagged recombinant enzyme was purified from the crude extract using NTA-Ni^2+^ agarose affinity chromatography and then further purified by size exclusion chromatography on a Superdex 200 HR 10/30 column. The SDS-PAGE analysis showed that only one single 38-kDa protein band was observed with 0.5 µg loading quantity of protein sample (Figure 3).

### Biochemical Characterization of the Recombinant DphB

The exact native molecular mass of the recombinant DphB was determined to be 37,743 Da by MALDI-TOF-MS. The molecular mass of the denatured recombinant DphB was determined to be 38 kDa by SDS-PAGE. These results indicated that the recombinant DphB was a monomeric protein. The pI of the recombinant DphB was estimated to be 5.59 by gel IEF.

**Figure 4:**
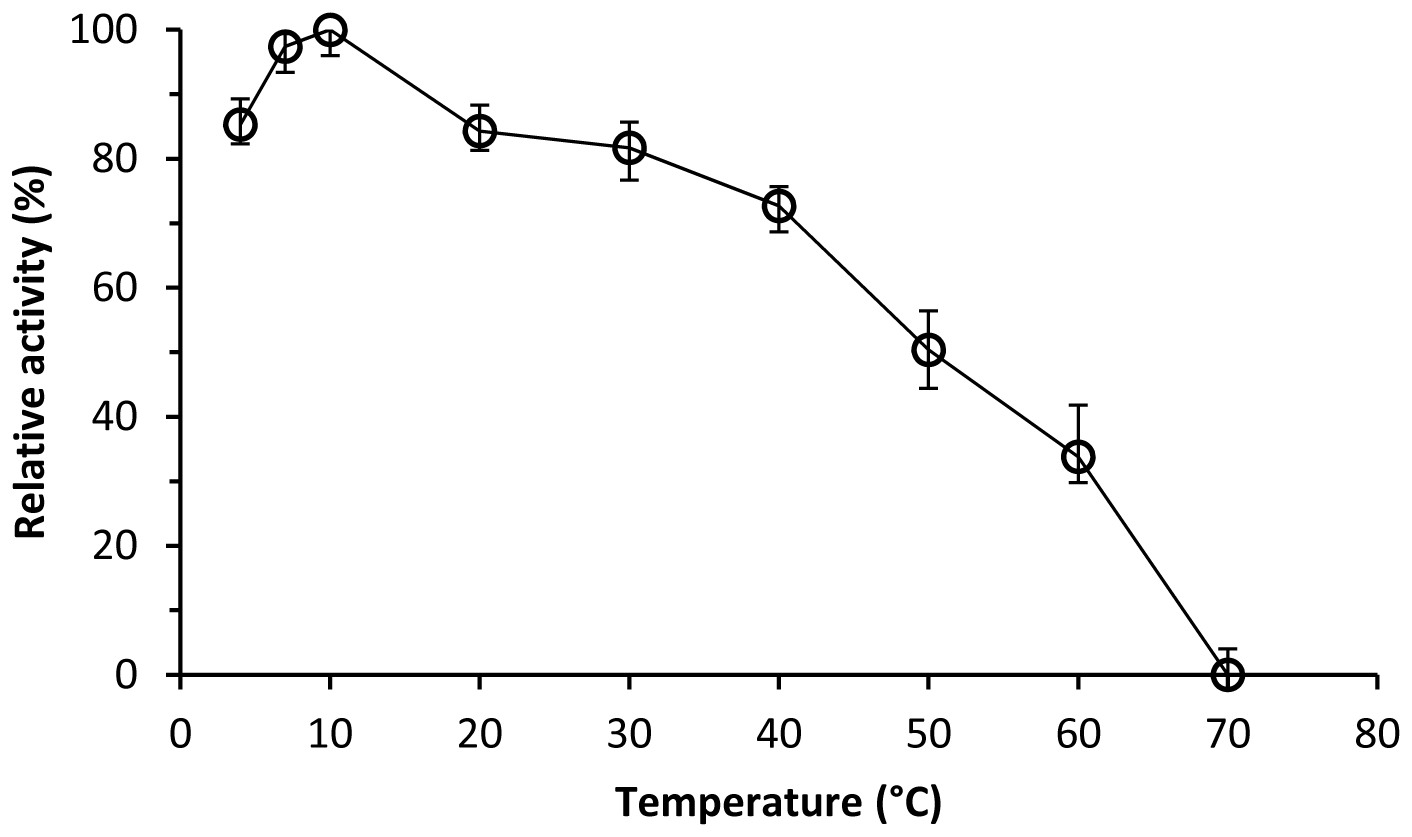
Effect of temperature on DBP hydrolytic activity of recombinant DphB.

**Figure 5:**
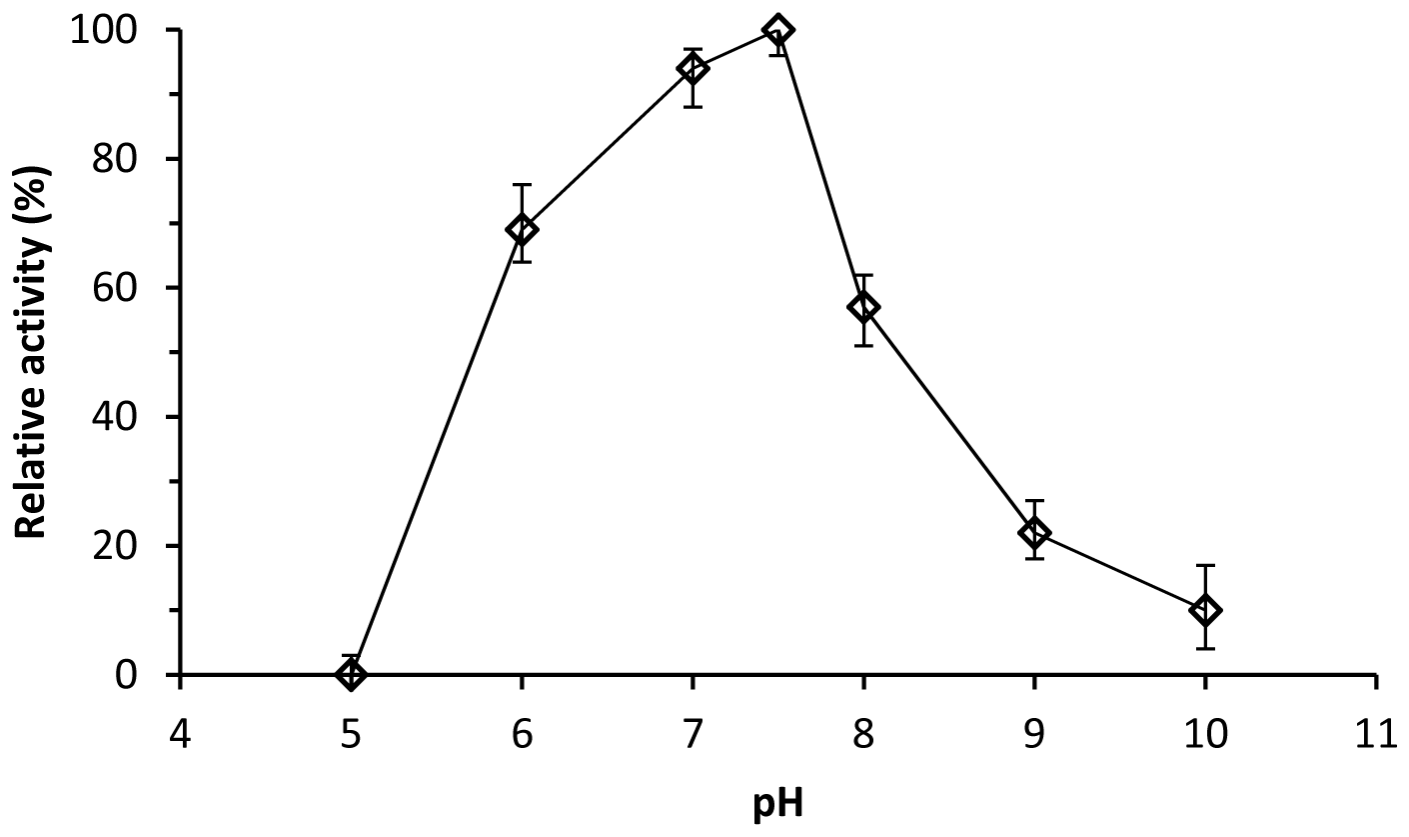
Effect of pH on DBP hydrolytic activity of recombinant DphB.

**Figure 6:**
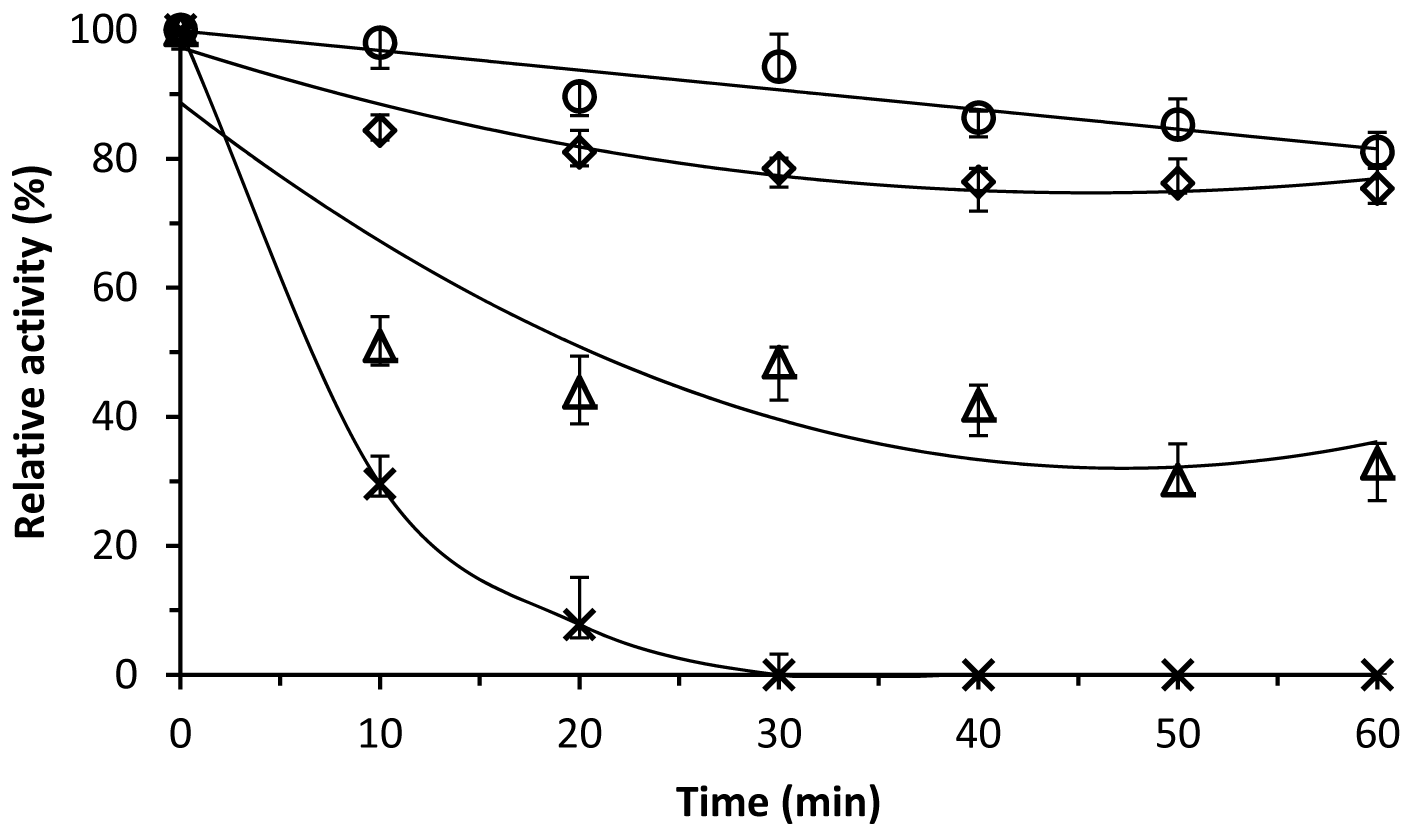
Thermal inactivation profile of recombinant DphB at 30°C (○), 40°C (◊), 50°C (Δ), and 60°C (×). DBP hydrolytic activity is expressed as a percentage of that at zero time in the standard assay at 10°C.

The dialkyl PEs hydrolase activity of the recombinant DphB was detected in a wide temperature range of 4 to 60°C, with an optimum temperature of 10°C (Figure 4), which suggested that DphB was a cold-active enzyme. When assayed at a pH range of 5.0 to 10.0, optimum activity of DphB was observed at pH 7.5, with approximately no activity at pH 5.0 (Figure 5). DphB was thermostable at temperatures below 40°C, and retained 90% of initial activity at 30°C over a 60-min period, but was rapidly inactivated at 60°C, with 30% of initial activity remaining after 10 min and no activity remaining after 30 min (Figure 6).

The substrate specificity of the recombinant DphB was then investigated with C_1_–C_7_ chain dialkyl PEs and monoalkyl PEs as the substrate respectively. DphB displayed the high specificity toward the medium chain (C_3_–C_5_) esters (DPrP, DBP, and DPP) (Table 1), with greatest catalytic efficiency shown toward DPrP (C_3_). Catalytic efficiencies directed toward DPP and DBP were 71% and 42% of that for DPrP, respectively. DphB failed to catalyze the hydrolysis of the short chain (C_1_–C_2_) esters (DMP and DEP) and the longer chain (C_6_–C_7_) esters (DHP and DHpP). This substrate specificity indicated that the accessibility of dialkyl PEs to DphB was dependent on the ester chain length of dialkyl PEs. Two reported mesophilic dialkyl PEs hydrolases, respectively, from *Acinetobacter* sp. M673 [15] and *Micrococcus* sp. YGJ1 [16], also displayed the similar ester chain length-dependence, with the medium chain dialkyl PEs as their most preferred substrates. But unlike DphB, they were able to catalyze the hydrolysis of the short and the longer chain esters, as DMP, DEP, and DHP. This result suggested that DphB might have a stronger ester chain length-dependence than the two enzymes mentioned above. It has been proposed that cold-adapted enzymes might trade off substrate affinity for catalytic velocity, seen as markedly high *K_m_* values and lower *k*_cat_/*K_m_* ratios [26], [27]. However, we did not observe the trade-off between affinity and catalytic velocity in this study. DphB, as a cold-active dialkyl PEs hydrolase, displayed a higher catalytic efficiency than the two mesophilic dialkyl PEs hydrolases due to its lower *K_m_*-values and higher *V_max_*- and *k*_cat_/*K_m_*-values in hydrolyzing DPrP, DBP, and DPP. Thus, it indicated that DphB did not seem to fit with this theory. DphB was unable to catalyze the hydrolysis of C_1_–C_7_ chain monoalkyl PEs, which clearly indicated that DphB was just a dialkyl PEs hydrolase, with specific activity for DPrP, DBP, and DPP.

**Table 1:** Kinetic parameters of the recombinant DphB for hydrolysis of seven various dialkyl PEs.

Substrate	Mean ± SD			
	*K_m_* (mM)	*V_max_* (µmol min^−1^ mg^−1^)	*k*_cat_ (s^−1^)	*k*_cat_/*K_m_* (mM^−1^ s^−1^)
DMP	/	0	0	/
DEP	/	0	0	/
DPrP	0.271±0.012	46.4±1.93	44.9±1.74	166±5
DBP	0.561±0.016	40.7±1.43	39.4±1.23	70±2
DPP	0.378±0.018	45.9±1.55	44.5±1.38	118±4
DHP	/	0	0	/
DHpP	/	0	0	/

/, not measurable; *K_m_* and *k*_cat_/*K_m_* could not be calculated due to specific activity data not being available.

### Identification of the Metabolites of DphB

The recombinant DphB may specifically catalyze the hydrolysis of DPrP, DBP, and DPP to the corresponding monoalkyl PEs. The hydrolysis of DBP typified this general transformation process. The metabolite was identified as MBP because of its identical retention time and mass spectrum as those of an authentic sample of MBP. Two weak quasimolecule ions at *m/z* 223.1 and *m/z* 467.1 represented [M+H]^+^ and [2M+Na]^+^, respectively, and the strong quasimolecule ion at *m/z* 245.1 represented [M+Na]^+^. The major fragment ions at *m/z* 118.0, *m/z* 139.9 and *m/z* 159.1 were identical to the proposed fragments using MBP standard as an example.

### Identification of the Proposed Catalytic Triad in DphB

Comparative sequence analyses using the BLAST program (http://www.ncbi.nlm.nih.gov/BLAST/) showed that a proposed catalytic triad was present in DphB as Ser159, Asp251 or Asp255, and His281 [23], [28] (Figure 2). In order to confirm the catalytic triad, we used alanine scanning mutagenesis to introduce four point mutations into its structure in these positions in which catalytic residues were expected. Our results showed that the CD spectra of the wild-type and four mutant proteins were essentially the same (data not shown) indicating that the backbone polypeptide chain of constructed mutants had a very similar conformation. It was concluded that the single point substitutions did not disturb the overall structure of the protein mutants. No activity was observed for the mutant enzymes Ser159Ala, Asp251Ala, and His281Ala even at very high substrate concentration (10 mM), while the mutant Asp255Ala still showed very weak hydrolase activity toward DBP, with *K_m_* of 20.3±1.17 mM, *V_max_* of 7.79±1.03 µmol min^−1^ mg^−1^, *k*_cat_ of 6.95±1.42 s^−1^, and *k*_cat_/*K_m_* of 0.342±0.023 mM^−1^ s^−1^, respectively. The activity of Asp255Ala was about 17% of the wild-type enzyme as deduced from the *k*_cat_ value. Thus, the catalytic triad residues of DphB were confirmed to be Ser159, Asp251, and His281.

The biochemical mechanisms underlying the hydrolysis of PEs in microorganisms have been intensively researched. One dialkyl PEs hydrolase gene from *Acinetobacter* sp. M673 (DBP hydrolase gene, JQ478494) and two monoalkyl PEs hydrolase genes, respectively, from *Gordonia* sp. P8219 (MEHP hydrolase gene, AB214635) and *Rhodococcus jostii* RHA1 (*patE*, Locus tag, RHA1_ro10206) have been cloned and sequenced. Sequence comparison revealed that the sequence of *dphB* gene shared no intensive homology with the three reported genes, and the deduced amino acid sequence of *dphB* also shared no significant identity with the three proteins. Thus, the results indicated that DphB was a novel member of the PE hydrolase family.

The PEs hydrolases play important roles in the decontamination of PEs and will be useful bacterial catabolic enzymes in the bioremediation of environmental pollution caused by these plasticizers. The ability of cold-active DphB to catalyze the hydrolysis of dialkyl PEs at low temperatures seems to offer a greater environmental application potential than those mesophilic dialkyl PEs hydrolases. Probably, the production of such recombinant enzyme may be ideal for bioremediation process in the field of wastewater treatment and in-situ degradation in contaminated cold environment.
